# Imagining a Safe Space: Australian Community Views About What Makes Crisis Mental Health Services ‘Safe’ and ‘Unsafe’

**DOI:** 10.3390/ijerph23010004

**Published:** 2025-12-19

**Authors:** Erin Stewart, Alyssa R. Morse, Heather Lamb, Helen T. Oni, Mel Giugni, Louise A. Ellis, Cassandra Chakouch, Dianna G. Smith, Scott J. Fitzpatrick, Michelle Banfield

**Affiliations:** 1 Independent Lived Experience Researcher, Canberra, ACT 2601, Australia; erin.stewart@outlook.com; 2 Centre for Mental Health Research, The Australian National University, Canberra, ACT 2601, Australia; alyssa.morse@anu.edu.au (A.R.M.); melanie.giugni@anu.edu.au (M.G.); dianna.smith@anu.edu.au (D.G.S.); michelle.banfield@anu.edu.au (M.B.); 3 Sonder, Adelaide, SA 5015, Australia; 4 School of Psychology and Public Health, La Trobe University, Melbourne, VIC 3086, Australia; 5 Australian Institute of Health Innovation, Macquarie University, Sydney, NSW 2109, Australia; louise.ellis@mq.edu.au; 6 Lifespan Health and Wellbeing Research Centre, Macquarie University, Sydney, NSW 2109, Australia; 7 Black Dog Institute, University of New South Wales, Sydney, NSW 2031, Australia; cassandra.chakouch@blackdog.org.au; 8 The ALIVE National Centre for Mental Health Research Translation, Canberra, ACT 2601, Australia

**Keywords:** suicide prevention, crisis, health systems, safety, lived experience, mental health

## Abstract

**Highlights:**

**Public health relevance—How does this work relate to a public health issue?**

- Safety is a primary concern of mental health services.
- Current conceptualisations of safety focus on risk management.

**Public health significance—Why is this work of significance to public health?**

- Novel insights into service ‘safety’ and ‘unsafety’ from mental health consumers.
- Experiences of safety shape levels of satisfaction and future service use.

**Public health implications—What are the key implications or messages for practitioners, policymakers, and/or researchers?**

- Risk management focus on safety is inadequate and harmful.
- Attention needed to spatial, temporal, and relational dimensions of safety.

**Abstract:**

Mental health services have an interest in maintaining psychosocial safety for consumers, carers, and staff alike. While much discussion around safety in service delivery pertains to the likelihood of patients engaging in damaging behaviours, we take the position that community attitudes towards safety offer more expansive, relational, and spatial definitions of safety. In a survey consisting of a mix of open and closed questions of 279 Australians aged 16–87 years, participants were asked to comment on their experiences of safety and unsafety in emergency mental health service use, as well as what they consider to be a safe or unsafe service. Applying a thematic analysis to the data, findings showed that emergency departments are not safe or appropriate for mental health consumers. Participants had heterogenous but largely consistent ideas about what made a service safe. Elements of safety mentioned by participants included a therapeutic orientation to time; service predictability; sensory dimensions of safety; and feeling understood. For some participants, notions of safety and unsafety dictated not only their satisfaction with services but overall likelihood of service use, thereby emphasising the critical importance of community attitudes towards safety in service design and delivery.

## 1. Introduction

As part of a wide-ranging evaluation of safe spaces, this study sought Australian community attitudes regarding the awareness, acceptability, and accessibility of these services to the public through an online survey [1]. Safe spaces are nonclinical, peer-led services for those experiencing emotional distress and/or suicidal crisis. The survey canvassed whole-of-community attitudes towards safe spaces, including but not limited to the views of consumers and carers. Unexpectedly, through four open questions in the otherwise quantitative survey, community members provided robust, vivid descriptions of what they imagined a safe mental health service to look like and visceral experiences of feeling unsafe in crisis service settings. We have delved into this unexpected well of data in order to answer the question, “How does the community imagine a safe space will look like?” and, relatedly, “what makes a service unsafe?” The present study reflects on these findings, and how participants defined ‘safe’ and ‘unsafe’ mental health services.

We are grateful that participants chose to share their experiences and views of what ‘safety’ and ‘unsafety’ means in a crisis context. Definitions of safety have tended, in the literature and in clinical practice, to exclude the lived realities of seeking crisis care, or imagining what seeking crisis care would—or could—feel like. In mental health care settings, notions of safety have often been conflated with notions of risk. Indeed, much funding, training, and staff time are dedicated to the management of potentially destructive behaviours, and to the implementation of generic guidelines to assess patient risk of suicide, self-harm, and danger to others [2]. Often, institutional definitions of safety run counter to consumer definitions. Consumers are more likely to consider iatrogenic harm, human rights abuses, poor continuity of care, staffing issues, and lack of choice as salient safety risks [3,4]. In assessing what constitutes safe practice, Seager writes [2], “we are far less focused and explicit about what good mental health might look like and even less explicit about what a safe service might look like.” A mismatch between staff and consumer understandings of safety and risk may lead to poor experiences for consumers. Those seeking refuge from the vantage point of a mental health crisis can be understood as being at risk of not having their needs met, particularly in mainstream clinical settings such as hospital emergency departments, a predominant means of seeking assistance for suicidal distress in Australia [5].

Even though it is common to present to hospital emergency departments during mental health crisis, there is a large body of research that shows mental health consumers often feel unsafe in these settings [4,6,7]. Environments are characterised by a cold, clinical feel [4], where consumers describe feeling alternately rushed and deprioritised as they wait long periods for medical attention [7]. They report feelings of not being taken seriously, being cast as burdensome, and being the subject of stigmatising attitudes [8]. Settings such as these also pose a risk to consumers of being involuntarily detained and subjected to traumatic coercive practices such as seclusion and restraint [8].

Environments that feel unsafe in these ways are incongruous with consumer needs [7]. Consumers seeking help tend to seek interpersonal connection and support catered to their specific circumstances [7]. Private, comfortable spaces are most appropriate for waiting and assessment [5]. Moreover, as a key risk factor of suicide and suicidal ideation is the experience of trauma, it is incumbent on mental health crisis settings to be trauma-informed [5].

Yet, consumers report seeking emergency support with the awareness that it may be a traumatic environment because they view emergency care as necessary. Their distress is felt to have life-threatening dimensions, and there are no known appropriate alternatives [9,10]. There is a clear need, therefore, to investigate consumers’ understandings of safety and how safe spaces may be provided to those who need them most. This study explores both participants’ felt experiences of safety and unsafety, and how they would define a safe (or unsafe) service.

The concept of a ‘safe space’ emerged from twentieth-century feminist discourse, which reframed notions of safety and unsafety away from formal risk assessment and towards iterative efforts to build relational and collaborative spaces. Drawing on the work of the Roestone Collective [11], a hallmark of such spaces is the celebration of diverse identities and that they allow inhabitants to negotiate differences, express need, and neutralise anxiety, threat, or fear. Under this framework, “a safe space is never completely safe”, but there exists a mutual commitment to working towards an inclusive sense of safety [11]. How such a space might manifest is contingent on the people present. Indeed, research on safe spaces in the context of emotional distress and suicidal crises has emphasised the relational, co-creative elements of building safety [12].

## 2. Materials and Methods

### 2.1. Study Design

This study is a subcomponent of the Co-Creating Safe Spaces project [1]. The study was a lived experience-led, co-created research project that used mixed methods to investigate the implementation, effectiveness, and sustainability of safe space models as genuine alternatives for people experiencing emotional distress and/or suicidal crisis who might usually present to the emergency department or choose not to access help due to past negative experiences. The current sub-study collected data via a self-report survey created in Qualtrics, specifically looking at the qualitative responses provided by participants. The majority of authors identify as having a lived experience of mental ill-health or suicide, either personally or as a carer or family member. Researchers with lived experience were involved in all aspects of the study, including design, data collection, and data analysis.

### 2.2. Recruitment

The study utilised a comprehensive recruitment strategy combining both social media and email outreach. A digital flyer was shared on platforms such as the Co-Creating Safe Spaces and ACACIA: The ACT Consumer and Carer Mental Health Research Unit Facebook pages and distributed nationally by email to over 600 contact points. Contacts included a diverse range of organisations, from small local volunteer groups to mid-size government and non-government organisations, peak consumer and carer bodies, lived experience networks, educational programmes, and research institutes across remote, rural, regional, suburban and urban areas. Contacts were invited to participate themselves and to distribute the flyer within their networks, newsletters, and social media channels. The flyers invited prospective participants to contribute to a community survey about safe spaces for people experiencing emotional distress and/or suicidal crisis, and to share their insights on new services and support-seeking preferences. This approach ensured a broad and effective distribution of the survey across diverse jurisdictions, platforms, and networks.

Participants were required to reside in Australia and be aged 16 years and over to complete the survey. Personal experience of suicidality was not a requirement for participation, and the survey was open to all who wished to contribute. The responses for this paper were collected between June and December 2023.

### 2.3. Data Collection

The Community Survey included a mix of open and closed questions. The current study reports on the open-ended questions relating to previous Emergency Department (ED) use and safety. Brief descriptive statistics have been included in this paper for context, including demographic statistics and statistics on the level of satisfaction participants had in relation to previous ED use. A separate paper will report quantitative survey findings.

To support safe participation in the anonymous survey, participants were clearly informed about question content on the participant information sheet. Participants could skip any question they preferred not to answer and could elect to skip survey sections containing potentially distressing content (e.g., items regarding distress and suicidality). Contact details for appropriate support services were linked on each survey page, and participants could elect to submit their data and exit the survey early at regular intervals.

Previous Emergency Department Use: Participants who had identified as having used an ED in the past were asked whether they were satisfied with the service using a Likert scale in which they could respond that they were either ‘Extremely,’ ‘Very,’ or ‘Slightly’ satisfied, neutral, or ‘not at all satisfied’ with the experience. This was then followed by the question: ‘Could you please briefly describe the main reasons for your response?’ (open text). Questions on satisfaction were only presented to a subset of our sample who indicated that they had either been to the emergency department for themselves or as a carer.

Safety: All participants, regardless of their lived experience, were given 3 open-item questions regarding safety and services. Items included ‘What makes a place or service feel safe for you?’, ‘What things do you consider before deciding if it is safe to visit a service?’, ‘What has been a ‘deal-breaker’ or stopped you from returning to services in the past?’, and ‘Are there any further comments you would like to make?’

### 2.4. Data Analysis

Open-ended responses were thematically analysed by the lead author (ES) and reviewed by the second author (ARM). The thematic analysis was conducted using Braun & Clarke’s [13] six phases. The first phase involved familiarisation with the data set, whereby ES became immersed in the reflections, stories, and opinions expressed by survey participants. In the second phase, coding and quotations were organised by ES into a Microsoft Word document in accordance with their content: for example, lack of physical safety in emergency departments (such as unsafe discharge or access to objects that can be used for self-harm); psychological unsafety in emergency departments (including waiting times, sensory overstimulation, lack of privacy, not being taken seriously); hallmarks of safe experiences in emergency departments; dealings with police/ambulance/non hospital services; psychiatric ward admissions; advocacy; follow-up and referrals; reform opportunities; the emergency department as a ‘last resort’; and refusal of future emergency department use.

At this point, initial themes were generated and the data were divided into four themes outlined in the Section 3: (i) spatial-temporal dimensions of safety, (ii) relational dimensions of safety, (iii) predictability as a marker of safety, and (iv) the stakes of safety. As the authors share a lived experience perspective with the survey participants, themes were intersubjectively co-created by both survey participants and the lead author. The overlapping experiences of the authors and participants shaped the narrative told in this research. At this point, key themes and relevant quotations were shared with co-authors in the form of tables of quotations and observations. This enabled co-authors to verify or contribute to the thematic interpretations provided of verbatim quotations. These tables were collaboratively honed into the text of the results, as co-authors shared their thoughts about what comments were most emblematic of the themes generated. In this process, Braun & Clarke’s [13] final writing up phase of analysis occurred alongside the phases of developing and reviewing themes and refining, defining, and naming themes. This ensured a collaborative process with co-authors having the opportunity to provide foundational input into the data analysis process without the labour intensity of widescale review of the raw data.

## 3. Results

### 3.1. Demographics

There were 279 participants in the survey who had a mixture of ages, genders, lived experiences, and cultural backgrounds. The majority of participants (62%) had previously visited the hospital or ED to seek help for suicidal distress, thoughts or action for either themselves, or someone they cared for. However, we were interested in how the whole sample conceived of safety and unsafety during crisis, regardless of whether participants could reflect on previous experiences of the ED. The demographic information of participants is included in Table 1.

Of those who had previously accessed the hospital or ED, over half (54%, n = 87) reported that they were ‘not at all satisfied’ and just over a quarter (28%, n = 44) were ‘slightly satisfied’, with few participants reporting more favourable levels of satisfaction (8.8%, n = 14).

### 3.2. Spatial-Temporal Dimensions of Safety

Sensory dimensions that make a space feel safe were described universally as “comfortable”, acknowledging some individual differences. One participant used the phrase “easy on the senses” to describe what they envisioned: “low light, quiet”, with “plush chairs, blankets, soft pillows, something to cuddle”, and “centred around my needs and preferences”. Considerations of lighting, noise, comfortable textures and surfaces, casual staff clothing, cleanliness, and breakout spaces to be alone or for private one-on-one chats were common among participants. Several participants further indicated they would feel safer in services that signalled acceptance and allyship such as through LGBTIQ+ and First Nations flags and indicators of accessibility (such as ease of wheelchair access). Exposure to natural environments, such as parks, beaches, bushland, trees, and plants, were desired. Many participants suggested that the availability of refreshments would also improve feelings of safety.

The way time was managed corresponded to sensory feelings of safety and unsafety. Common experiences in EDs and other crisis settings involved long wait times in a busy environment. This invoked a feeling of others rushing around while the consumer was distressingly still. Participants estimated that they had spent between 3 and 8 hours waiting to be seen in EDs with some reporting not receiving specialised psychiatric help for days. Participants further said that once the waiting was over, they were rushed out of the service. Participants felt that there were limited opportunities to be truly listened to.

“The wait times are long, there is nowhere private to wait, staff treat you like an animal, inconvenience or similar, staff patronise you to make you “go away” or “get over it” and contribute to distress in patients and our failing mental health system”.

The discomfort of waiting was reported to be amplified by the sensory dimensions of the waiting area which “lacks any warmth, comfort or aesthetics that would contribute to the patient improving their mental health”. One respondent reported leaving the ED after three suicide attempts and waiting for three hours for help, because they could not bear the sensory environment: “I was already beyond my tolerance level for being in a crowded area, I couldn’t stop shaking and felt on the verge of a panic attack. I left the ED without getting any help”.

In contrast, participants expressed gratitude when they were given adequate space and time to develop a sense of calm, away from the overwhelming waiting room environments. Small gestures, like when a triage nurse “acknowledged [the loved one’s] pain and suffering and offered a quiet room to sit” made “such a huge impression”. Participants also offered ideas for potential reform, such as separate waiting rooms for people experiencing mental health concerns, and/or quick transfer to psychiatric wards for triage.

### 3.3. Relational Dimensions of Safety

Participants identified relational elements of safety including the provision of information about individual rights; consensual, person-centred and person-led approaches with a strengths and values focus; and communication with “no expectation to talk if I’m not ready”. Support people would be welcomed into a safe service, and indeed, there would be additional support available for carers, family, friends, and kin.

Participants described the skills and qualities they seek in staff: non-judgmental, empathic and compassionate listeners who have the time to talk and “meet me where I’m at”; skilled in reducing distress; trustworthy, trauma-informed with an understanding of the social model of disability; staff who recognise their power and privilege while being able to acknowledge and respect consumer expertise. Continuity (seeing the same person each time) was welcomed. Good follow-up procedures were also noted as hallmarks of satisfactory encounters.

In contrast, in emergency settings, participants experienced dehumanising treatment. Participants frequently compared their experience to being imprisoned or regarded as a burden, an animal, or “sub-human”. One described the “judgement and shame…being locked in a room guarded by security, treated like a prisoner, no compassion”. Several participants also highlighted their experiences of coercive practices such as physical and chemical restraint as well as non-consensual seclusion and hospital transfers.

Damaging comments and stigmatising staff attitudes were reported by multiple participants, with one reporting that a paramedic told them that “I was wasting their time and I should have just killed myself”. Another person, after telling staff they were suicidal, reported that the staff responded: “No you’re not. If you were going to you would’ve by now”. Other comments, such as the person was “wasting a bed” were also shared.

Less physically visible dimensions of mental health were reportedly taken less seriously than physical health. Multiple participants expressed disappointment in their experience that if physical health was assured, then ongoing emotional distress was seen as insignificant. One person reported being discharged after their pathology report “said [I’m] ok”. Another had their self-harm wounds promptly tended to but left the hospital after finding out that receiving mental health support would take five hours of waiting.

“People with mental health conditions including having suicidal ideation are often not taken seriously because they are not bleeding, having a heart attack or have any broken bones”.

Where survey participants did have positive experiences within EDs, certain hallmarks of quality care were evident in responses. Empathy and respect by staff were key factors, alongside qualities of friendliness, patience, and validation.

“[I was] very satisfied with the personalised interactions and the genuine care and respect for me being an older homosexual. They even respected my carer… I was there so long they even gave me and my partner water and a sandwich. I felt safe there for the first time in years and did not feel I was being ignored or hurried. Things can change”.

Multiple participants reflected that it would be desirable to wait alongside designated peer workers in order to attain a relational sense of safety. Indeed, lived experience staffing was endorsed overall as a feature of a safe service. Some participants only wanted to engage with peers or with services that were at least peer-led, while others were comfortable with multi-disciplinary teams.

While a peer workforce was considered largely desirable, there were certain concerns that could compromise safety. One respondent said they had experienced peer workers becoming overwhelmed by their story, so professional boundaries are an important aspect of safety. Privacy issues may also emerge, as per responses, where a peer worker needs to seek care themselves. Another respondent, a clinician, expressed some concern that peer-led services could “simply validate participants [and] not challenge them enough”. However, “validation” was an important theme for many participants, suggesting there is a need to balance expertise and professionalism with friendliness, freedom, and choice.

### 3.4. Predictability as a Marker of Safety

Aside from the uncertainties associated with waiting for unknown lengths of time, other uncertainties jeopardised the ability of services to feel safe. The survey data showed that uncertainty clustered around aspects of the model of service as well as unknown characteristics of staff.

In terms of the model of service, participants showed apprehension towards the loss of control of their own decision-making and/or private information. The threat of distressing encounters such as confidential information leakage, violence, aggression, being subjected to coercive practices, or even assault were considered “dealbreakers”, rendering a service unsafe.

Predictability, on the other hand, was highly prized as a characteristic of safety. As one participant surmised:

“I would be more likely to visit a place or service if I knew more about what would happen once I got there (e.g., who would I meet/talk to, what would the environment be like, how long would I be there, would there be an expectation for me to talk)”.

Knowing that consumers will be able to maintain autonomy over their decisions through their interactions with the service was an important safety measure. This involved being able to control how, when, and whether consumers interacted with others, including staff, and what consumers could do to occupy their time (reading was expressed as a preferred activity for some participants).

Participants further sought clear information about confidentiality, record protection, and clarity about record-sharing. Participants expressed discomfort at the lack of privacy engendered by clinical settings where consumers were forced to “speak loudly over counters or through plastic barriers to get assistance”, or where “more often than not, the most ‘privacy’ you get is the clinician closing the curtains”.

Continuity of care, including familiar staff, consistent opening hours (preferably “24/7” availability), and being able to orient oneself to the space before using the service were also valued. Participants suggested that they would benefit from opportunities to visit the space, talk to, or message staff prior to service use “so I can know what to expect”. One participant would “look online to see staff profiles and get a sense of the services approach (e.g., if they are genuinely non-clinical)”. Some suggested tours or virtual tours where you could see the space, read about the process of entering the space, and create representative expectations of the space. Participants also reported drawing on “word-of-mouth” recommendations for services to ascertain a sense of trust.

Advocacy was a means for some to feel a degree of control in emergency situations. While we would hope that services are safe for all people, for some it was only achieved where there was sufficient informal support or skills in self-advocacy to overcome barriers and negative experiences. Two participants expressed that their skill in self-advocacy (for example, stating “I am at risk to myself”) empowered them to express their needs in a way for staff to understand and “respond”. Another reflected, “We were lucky we were able and willing to advocate, push, support and facilitate action for our loved one—or the alternative would have been suicide”. In contrast, some participants reported evoking medical language and signals of wellness to take control of an emergency situation that felt out of control. Some spoke of “faking” or “masking” to seem “well”, “so I can get home”.

For some participants, for whatever faults emergency services may have, there was a feeling of safety in obtaining crisis care. This was because the sets of rules and procedures governing care felt more predictable than dealing with crisis outside of professional expertise. As one respondent, a carer, wrote:

“Once you manage to get someone in need of help into the system even by having to involve the police you breathe a sigh of relief as you know more can be done for them to help than you are able to do”.

### 3.5. The Stakes of Safety

Multiple participants described inadequate care and unsafety in EDs, such as being pre-emptively sent home without treatment or follow-up for ongoing suicidal thoughts.

“I was sent home to WALK HOME while suicidal. I’ll never, ever forget the shock of one minute being in an emergency department waiting for psych triage, telling them my distress, and them not having any regard for me walking the 5 km back to my home, alone”.

Survey participants reflected on either themselves or a loved one being put in situations where they could have, or did, come to irreparable harm. In one case, a respondent reported being given back a bottle of pills they had earlier used in their suicidal overdose by a nurse; they subsequently used it to overdose again. In other cases, participants shared that they had self-harmed because they understood that they would not be taken seriously if they presented with only suicidal ideation.

Many comments reflected a reluctance to attend EDs with some seeing it as an option of “last resort”. Although generally unsatisfied with the care they received in EDs, some participants did acknowledge that it kept them physically safe. A consumer described their own deliberations:

“It’s a draining experience presenting to an ED—both mentally and physically. I avoid it at all costs now… In saying that I do recognise and also acknowledge that there are times, and will be times, when I will be so acutely unwell that I will need to attend an ED and possibly be admitted into the inpatient unit, so it does have its place and a role to play”.

For some participants, notions of safety and unsafety dictated not only their satisfaction with services but overall likelihood of service use, thus emphasising the critical importance of community attitudes towards safety in service design and delivery. Participants expressed a range of preferences for alternative services such as peer support groups and services, crisis phonelines, and direct support from family, friends, kin, Elders, or even colleagues.

While some thought of EDs as necessary, albeit uncomfortable, others found them to be unacceptable. One participant said they would not go to a service that was even in close proximity to a hospital and multiple participants said they would not go to a service that reminded them of hospitals. Some participants who had reported unsafe experiences expressed they would never use the services again. For example:

“The way I was treated traumatised me even more to the extent I will never voluntarily go there for support. I am too fearful. I believe poor communication and system processes, disempowerment, lack of empathy, understanding and kindness toward my family and I only increased our emotional distress”.

The lack of willingness to attend services was acknowledged by participants to have high-stakes repercussions. One respondent said: “I’d be better off killing myself than seeking help”. Another respondent, a carer, witnessed the death of their son because of an unwillingness to attend the ED again after two experiences where the “wait time was disgraceful for someone in distress…[he] ended his life in our home”.

## 4. Discussion

Participants who had experience with mainstream emergency service use overall were unsatisfied with the experience and had many safety concerns. Elements of safety included a therapeutic orientation to time, neither weighed by long periods of waiting nor rushed; predictability in terms of the mechanics of the service; the preservation of personal choice, as well as the quality of the staff; attendance to matters of sensory safety; and the provision of access intimacy where articulations of distress would be understood. The stakes of safety in service delivery, as expressed by survey participants, was high. Participants suggested that they would avoid unsafe services, even to the point of personal detriment, and that unsafe services compelled them or loved ones towards significant self-harm.

These findings are distinct from but align with previous studies regarding safety in emergency settings. In a qualitative study of 31 in-depth interviews with mental health consumers, Roennfeldt and colleagues [14] identified several elements of ‘embodied safety’ and that consumers seek holistic, human-to-human care that cultivates a sense of meaning from crisis. In a safe context, crisis may be transformed from being a risky state to a normalised aspect of being human. Another focus group study of seven individuals with lived experience of mental ill-health emphasised a sense of belonging as a hallmark of safety, as experienced through accessibility, symbolism (for example, staff uniforms were seen as symbols of unsafe hierarchy), and the presence of professional peers [6].

The findings of these studies align with humanistic approaches to time, relationships, and space reflected by participants in the present study that will form key elements of this discussion. Interestingly, our study showed that these elements of safety were reflected across larger sample sizes and are shared among people without lived experience of ED use, or of mental ill health, or of caring. These reflections can be understood as highly salient to this wider population. After all, it would be expected that interview and focus group participants would be able to articulate dimensions of safety when questioned deeply on it by researchers. In the context of this study, imaginings and criticisms of safety were invoked spontaneously in optional survey responses. A vision of what safety looks like was close to hand for participants without being drawn into reflective discussion.

Our findings also had interesting parallels with a recent study on developing accessible autistic ‘SPACE’, where SPACE is an acronym for ‘Sensory needs, Predictability, Acceptance, Communication and Empathy’ [15]. These elements were identified by survey participants as an important part of safety. The similarities found may reflect a high prevalence of neurodiversity among participants. Indeed, it has been established that autistic people are more likely to experience mental health distress and suicidality, and so crisis safe spaces must also be informed by autistic experiences [16]. In addition, this finding may point to designing for different sensory needs as a fundamental aspect of universal service design. That is, where people with diverse disabilities, trauma histories, age groups, and other access needs are incorporated into service design [17].

Survey participants reflected on a diverse set of experiences around mainstream emergency mental health services and safety. Even individual participants reported different experiences over multiple presentations to EDs, psychiatric wards, and other kinds of emergency care. Yet, the overall rubric the participants, in tandem, created for ascertaining safety and unsafety was largely consistent. It should be noted that the sample contained limited representation from some populations (e.g., men, CALD communities) and there would be value in exploring the experiences of these groups in greater depth. These results give an indication of qualities of crisis services that would inform overall likelihood of engagement, as well as likelihood of ascertaining comfort, healing, and de-escalation.

While these findings have emerged from a wide community survey, it is also important that safety be conceptualised within specific cultures and communities, and by the people inhabiting the space. Many participants in the study referenced identity markers—such as neurodivergence, LGBTIQ+ and First Nations status—in open-item questions, indicating that the unique intersectional nature of both an individual and the space is a key consideration of safety. Adaptively including people is portrayed as one of the tasks of a safe space. As per feminist constructions of safe spaces, safety is not an achievement per se, but an ongoing, relational dialogue requiring renewal and care over time [11]. We have shown through other research investigations that co-production—where collaboration exists between service stakeholders and centres lived experience—is highly effective at designing approaches that truly fit the needs of all stakeholders [12].

### 4.1. Therapeutic Orientation to Time

A frequent source of consternation with EDs was waiting times. Comments about wait times largely conform with Australia-wide data on the experience of presenting to EDs. In 2023–2024, the median time between a mental health emergency presentation and the commencement of clinical care was 21 minutes [18]. Such ‘wait time’ data, however, provides a limited phenomenological view of the reality of waiting. The commencement of clinical care means that a clinical pathway or protocol is established for a consumer. However, much more waiting often follows. The median length of stay in an ED for a mental health consumer was 5 hours and 3 minutes, with 10% of consumers having a length of stay longer than 18 hours and 52 minutes [18]. Both wait times and stay times are longer for mental health consumers than for people presenting with other health issues [18]. After an ED visit, 52% of mental health consumers depart without being admitted or referred to another hospital [18]. This makes any suggestion of symptom relief resulting from an ED presentation highly dubious.

Waiting is often a pre-condition of mental health care, whether in emergency department settings (where one sits in a waiting room) or in the community (where one may be placed on a waiting list). In emergency department settings, long waits are associated with higher mortality rates and increased risk of aggressive or violent behaviour within the ED [19]. Emotionally, waiting for care (such as being put on a waiting list) has a dose–response relationship with heightened distress, increased uncertainty, anger, frustration, and reduced self-esteem [20]. Long waits reduce trust in the system and quality of care, and are associated with lower engagement rates [20].

While waiting was considered unsafe, at the same time, so too was the feeling of being rushed or the prevailing condition of ‘busyness’ within service settings. Participants consistently identified the need for time and space to sit with one’s own feelings, and the choice about when and how to speak about themselves and their distress. The co-occurring need for speeding up (of triage and service delivery) and slowing down (of timelines surrounding talking about distress) can be understood through the disability studies lens of ‘crip time.’ In a society where access barriers are prevalent, crip time highlights the complexities and importance of emergent rhythms that enable individuals to safely navigate spaces, work with (rather than against) limitations, and recover from crises [21]. Crip time can also integrate ‘time travel’, such as navigating the immediate presence of previous traumatic experience, or contending with being treated as a child though you are an adult [22]. Neurodivergent people can experience a disorientation in time, known as ‘time blindness’ that can make it difficult to estimate time; and can make time feel like it is going overwhelmingly fast or onerously slow [22]. Crip time does not map nicely onto the routines and priorities of institutionalised spaces such as hospitals. In such spaces, time is defined around external key performance indicators, the demands of shifts, and the dynamics of supply and demand. Crip time, by contrast, is defined around idiosyncratic internal needs, for instance, for reassurance, holding, care, comfort- and trust-building. A safe space is one that accommodates time in flexible ways.

### 4.2. Predictability

When a person presents to a service for crisis care, they take on risks that elements of treatment will become out of their control. Participants talked about some of the most serious examples of unpredictability—where police responded to their distress, for example, or when non-consensual or coercive practices took place. By contrast, it was a relief to know what will happen, that the service will be open, that you will know the staff you will interact with, and what is expected of you in terms of disclosure and interaction. You know that you will be welcomed there.

There were some tensions around participants’ imaginings of predictable services in relation to funding limitations, and indeed, some degree of contradiction within these requirements. It would be difficult to guarantee continuity of care, for example, at a 24 hour service with varying staffing and shifts. Nonetheless, this ideal of familiarity and consistency is an aspiration a crisis service could converge upon. Other aspects of predictability could be realised straightforwardly. Some participant suggestions around orienting to the space through early interactions and tours were reminiscent of ‘social stories’; efforts to make spaces accessible by providing guidance on navigating them online, previewed before a person first enters. An example of a social story is provided by Hobart Airport, which contains tips and videos on moving through the airport, from parking to boarding the aircraft [23]. The effectiveness of social stories has primarily been studied on autistic children; however, there is evidence of their ability to improve situational predictability and transition management, alongside reducing anxiety [24]. Social stories, or similarly co-produced resources for managing service unpredictability, could be further investigated for the purpose of providing spatial safety.

Characteristics of staff were also discussed by participants in terms of safety. As some participants noted, there is a view that presenting at EDs requires accepting uncertainty about who will be assigned to your care. Worries such as staff being under-trained, having stigmatising attitudes, having or enacting discriminatory beliefs, or staff ‘going through the motions’ in an uncaring or indifferent manner characterised feelings of unsafety. This belief is in keeping with a UK-based study of emergency department staff members own assessments of barriers to safe care where a lack of training and confidence regarding mental health matters, burnout, and poor institutional support and culture were found to jeopardise consumer safety [25].

For participants who have been subject to ‘worst case scenarios’ like coercive practice, the range of possible treatment outcomes may be too broad to apprehend, particularly in a crisis situation. In these settings, trust is not guaranteed, although vetting through the form of positive-word-of-mouth from trusted peers would promote feelings of safety. Self-referral to psychiatric wards has also been found to be a promising means of avoiding the unpredictability of psychiatric admissions through emergency departments [26].

### 4.3. Sensory Safety

Alongside dimensions of model of care, quality of care, and competence of staff, many participants evoked sensory dimensions in their understanding of safety. In a review of studies of sensory dimensions of mental health services, sensory-appropriateness was found to be associated with reductions in consumer distress [27]. The authors note that sensory design is often used in an effort to reduce cases of seclusion and restraint [27]. Findings from this study showed that sensory features of the environment also featured heavily in participants’ accounts of what it means for a space to be safe.

While consideration of sensory design may help achieve desirable clinical outcomes, this research shows that sensory design forms part of whether some consumers will use the service at all, and whether they feel safe in using it. For some, a safe service by definition cannot look like a hospital. A surprisingly consistent portrait was painted by participants of a safe environment involving tactile comforts, pleasing or limited scents, auditory musicality or quiet, warm, soft, and/or natural lighting. Participants wanted the ability to be alone or with others, and the availability of refreshments and distractions. Naturalistic scenery was frequently suggested, a clear juxtaposition to the fluorescently lit, windowless, airless, stainless steel and tile-clad, bleach-scented hospitals.

Some desirable sensory provisions conflicted between individuals. Thus, services may need to accommodate different sensory needs within a location, perhaps by setting up different rooms with different levels of sensory input—such as a darker, quiet, minimalist space separate to a brighter space with music and stimulating artworks and diffuser scents. Sensory modulation—the ability to increase and decrease the strength of lighting, music, scent, and so on—could also facilitate different needs at different times.

### 4.4. Access Intimacy

Many participants reflected on difficulties with articulating distress, and having that distress heard, particularly in noisy, busy environments. Several participants commented that as mental health concerns often do not come with attendant visible symptoms, and as pathology and other tests indicate presenting individuals are ok, it is difficult to be adequately seen. Participants reported feeling as though they were a burden, that they were highly stigmatised and on the receiving end of confronting comments, and that their presence constituted a waste of resources.

As multiple participants reflected, it may be possible to communicate the meaning of distress through deliberate individual advocacy. This requires psychoeducation and/or learning a specific vocabulary that is more likely to be heard in clinical settings. For example, the sentence “I am at risk to myself”, used by one respondent, has a particular clinical meaning and is assigned a particular risk profile, which will more likely lead to admission. The adoption of clinical language allowed the speaker to control their distress’s reception, rather than relying on staff to infer a level of risk that would result in hospital admission or other forms of care desirable to the speaker. This language-learning also seemed to work in the opposite way, as a means of avoiding unwanted admission.

These forms of self-advocacy may allow consumers to form a greater sense of choice and empowerment as they navigate through mainstream emergency services. However, it also compels them to retain a high level of interpersonal engagement and effort, which may be beyond the means of many experiencing crisis. Consumers are advantaged where they have the relevant education and training to understand and use medical jargon. Such jargon may be compelled in quick measure, through technical questions about diagnosis, names of medications and dosages [8]. This type of language ventures outside of more natural ways of describing distress, which often require more time to articulate—and to hear—than what an ED can often afford. In the context of urgency, rudeness is rendered allowable—it is not unusual for consumers to overhear staff talking about their distress in the third person, as though the consumer is not there [8].

Sometimes words are not enough. It was disappointing to read of accounts where individuals undertook self-harm in order to be taken seriously. This finding is echoed in other work, where consumers reflect that the invisibility of distress adds to a feeling of inarticulability that evades appropriate risk assessment unless accompanied by an act of self-harm [28]. Despite efforts to orient mainstream emergency services around risk reduction, restricted forms of meaning-making and clinical framings of how stress is understood and responded to in these settings can create perverse incentives to heighten one’s level of assessed risk.

When describing elements of safety, participants mentioned that services would feel safer if consumers were considered to already hold self-expertise, where they could be themselves without having to justify their reason for being there, and where the service would meet consumers where they were at. There would be no need for the artifice of advocacy, which would enable the consumer to relax into the space. To this end, many participants suggested that the presence of peers (or peer-led services) characterised a safe space, although few described why peer workers felt safer to them. Part of the reason may lie in the concept of ‘access intimacy.’ Access intimacy describes situations where others adapt to one’s needs with little fanfare or conflict. Having your needs fulfilled involves simply asking for them—without the need to advocate or fight for them—or having them fulfilled without even needing to ask in the first place. As Mia Mingus [29], who coined the term describes it: “Access intimacy is that elusive, hard to describe feeling when someone else ‘gets’ your access needs… Sometimes it can happen with complete strangers, disabled or not, or sometimes it can be built over years. It could also be the way your body relaxes and opens up with someone when all your access needs are being met.” Currently, no scholarly work appears to exist on access intimacy as a potential benefit of peer work; however, reported benefits pertain to similar concepts such as empowerment, working alliance, experienced stigma, and social network support [30].

Qualitative investigations of the role of peer workers within EDs show that such access-intimate support is a promising direction for reform. Peer workers can effectively offer emotional support, deep listening, comforts such as blankets and meal trays, and de-escalation [5,7]. To this end, McIntyre and others [10] explore how individual support workers—including peer workers—helped those in crisis safely navigate EDs and potentially avoid their use in the first place through de-escalation techniques. However, as Brasier et al. [7] point out, it is not clear from the data if reform of existing EDs to include peer workers would be more or less effective than offering peer-led alternatives.

## 5. Conclusions

Findings from this study showed safety as spatial, temporal, relational, and characterised by predictability. Achieving service safety is necessary to engage consumers and carers with adequate support in the event of a personal crisis. A risk-management lens of safety that centres on the management of potentially destructive behaviours has been shown by this research to be inadequate and harmful. It does not ensure that consumers and those supporting them feel safe. Mental health services should work towards mutual understandings of safety, taking a collaborative, relational approach to establishing the feeling of safety. Our research has shown that such feelings would be enhanced through a flexible approach to time, improved predictability, sensory safety, and access intimacy. The inclusion of appropriately trained and supported lived experience staff was endorsed overall as a feature of a safe service. However, a co-production approach should be utilised by individual services to cater to the needs of those inhabiting specific spaces.

## Figures and Tables

**Table 1:** Participant Characteristics, N = 279.

Age (M, SD) *	47.2 (15.0)
Gender (n, %) ^+^	
Female	214 (77)
Male	47 (16.9)
Non-binary	11 (4)
Another gender	4 (1.4)
Prefer not to specify	2 (0.7)
Primary perspective (n, %) ^+^	
Personal experience of suicidal thoughts or actions	144 (51.8)
Family member, friend, support person or informal carer	48 (17.3)
Professional	67 (24.1)
General public or other	19 (6.9)
Identifies as CALD and/or speak a language other than English (n, %)	
Yes	31 (11.1)
No	248 (88.9)
Previously visited the hospital or Emergency Department to seek help for suicidal distress, thoughts or action for yourself or someone you care for (n, %) ^+^	
Yes	163 (61.5)
No	102 (38.5)
Participant location at time of survey (determined by postcode) (n, %) ^+^	
Australian Capital Territory	48 (17.3)
New South Wales	89 (32.1)
Northern Territory	6 (2.2)
Queensland	36 (13)
South Australia	28 (10.1)
Tasmania	21 (7.6)
Victoria	21 (7.6)
Western Australia	28 (10.1)

^+^ Percentages are a valid percent of completed responses for each characteristic. * Age range: 16–87 years.

## Data Availability

Data generated and analysed during the study are not publicly available due to privacy or ethical restrictions but are available from the corresponding author on reasonable request.
